# Human immunodeficiency virus status in malnourished children seen at Lagos

**DOI:** 10.1371/journal.pone.0200435

**Published:** 2018-10-04

**Authors:** Edamisan Olusoji Temiye, Oluwafunmilayo Funke Adeniyi, Iretiola Bamikeolu Fajolu, Ann Abiola Ogbenna, Taiwo Augustine Ladapo, Christopher Imokhuede Esezobor, Adebola Olumide Akinsulie, Cecilia Abimbola Mabogunje

**Affiliations:** 1 Department of Paediatrics, College of Medicine, University of Lagos/Lagos University Teaching Hospital, Idi–Araba, Lagos, Nigeria; 2 Department of Haematology, College of Medicine, University of Lagos/Lagos University Teaching Hospital, Idi–Araba, Lagos, Nigeria; 3 Massey Street Children’s Hospital, Lagos Island, Lagos, Nigeria; World Health Organisation (Head Quarters), SOUTH AFRICA

## Abstract

**Introduction:**

Human immunodeficiency virus and protein energy malnutrition are still prevalent in Nigeria and the occurrence of the two conditions together confers a poor prognosis. The aim of this study was to determine the current categories of malnutrition amongst under-5 children in Lagos, document their HIV status and determine any peculiarities in the clinical features, haematological and some biochemical profile in these children.

**Methods:**

The study was a cross-sectional study conducted at the Paediatric departments of the Lagos University Teaching Hospital and the Massey Street Children’s Hospital, both in Lagos, over a 6-month period. All the subjects had anthropometry, HIV testing, full blood count and serum proteins done. The factors associated with HIV status were determined with the logistic regression analysis.

**Results:**

Two hundred and fourteen (214) malnourished children ≤5 years, including 25 (11.7%) with HIV were recruited in the study. Among the study participants, 150 (70.1%) and 54 (29.9%) had moderate and severe malnutrition, respectively. Fever, cough and diarrhea were the most common symptoms in the study participants. The haematological indices were comparable in the two groups, the serum globulin levels though higher in the HIV infected group was not statistically significantly different from the non-infected group.(p = 0.66). None of the factors explored on multivariate analysis was able to predict the occurrence of the infection in this cohort.

**Conclusion:**

Malnourished children remain a high risk group for HIV infection and the prevalence of the infection obtained in this group of children is still unacceptably high. Discriminatory features between malnutrition and HIV remains difficult. The presence of hyperglobulinaemia on laboratory analysis in a malnourished child may heighten the suspicion of possible underlying associated HIV infection. Screening of malnourished children for HIV infection and further longitudinal studies on malnourished children with HIV is advocated

## Introduction

Human Immunodeficiency Virus (HIV) infection and its attendant problems remain a threat to global health, more so in the sub Saharan continent where malnutrition is also prevalent and the paediatric age group remain the most affected vulnerable group.[1] In developing countries, malnutrition contributes significantly to 50% of childhood mortality either directly or indirectly.[2] The mortality that occurs from the malnourished state is significantly related to the body growth parameters, namely the weight for age, weight for height and height for age. [2, 3] Severely malnourished children with z scores of the weight for height standard deviation score three or more below the mean values (-3 SD)less than 3-standard deviation from the mean values (-3SD) are at a 10- fold risk of a higher mortality compared to children with SDS a z-score higher or equal to 1.[2,3,4]

Malnutrition and HIV infection are believed to be intertwined in a vicious cycle that leads to immunosuppression. as HIV infection directly causes immunosuppression, and through various mechanisms including anorexia and repeated infections causes malnutrition. On the other hand, malnutrition, by impairing both innate and specific immunity predisposes to immunosuppression. Although, either HIV infection or malnutrition is associated with increased odds of mortality, together, both conditions significantly increase the risk of deaths. This synergistic effect of HIV infection and malnutrition on mortality creates a need to screen for HIV infection in malnourished children and malnutrition among children with HIV infection. Furthermore, HIV increases the vulnerability to malnutrition while the latter lowers immunity and enhances vulnerability to HIV and disease progression in HIV infected children. [5–7]The clinical features of severe malnutrition and HIV/AIDS are known to overlap in young children which may affect the accurate diagnosis of presumptive HIV infection in resource poor settings where HIV testing facilities are still inadequate and the two conditions often coexist. Malnutrition, although mainly attributed to nutritional deficiency, may be multifactorial in origin and the malnourished state which is usually accompanied by some degree of immunocompromise predisposes to infections generally and this does not exclude the HIV infection. The World Health Organization (WHO) actually classifies wasting as a Stage 3 AIDS condition[8–9] and thus distinguishing malnutrition from the former clinically sometimes can be sometimes difficult thus it is worthwhile screening malnourished children for HIV infection.

The prevalence of HIV in malnourished children has varied over the years, depending on the characteristics of the group of children studied. In 1990, Fischer et al [9] documented a prevalence of 3% in south West Nigeria. However, other reports gave a higher prevalence in this group of children, ranging from 8.6% in Niger Republic [10] to 12% in Central African Republic.[11] Most of these reports were in severely malnourished children. In 2014, some authors from Northern Nigeria, i,e., Kano, documented a prevalence of 7.8% across the various degrees of malnutrition. These variations were largely attributable to differences in degree of malnutrition and maternal HIV prevalences in the different groups. However, in south western Nigeria there are no current studies to reflect prevalence of HIV status in malnourished children. Thus, this study aimed to determine the prevalence and categories of malnutrition amongst children attending two health facilities in Lagos, the Lagos University Teaching Hospital (LUTH) and the Massey Children’s Hospital, and document the HIV status of these malnourished children. In addition, the study aimed to describe any peculiarity in the clinical features, haematological indices and some biochemical indices in these children.

## Materials and methods

### Data collection

The study was conducted in two health facilities (Lagos University Teaching Hospital and Massey Street Children’s Hospital) in Lagos over a 6-month period (October 2016-January 2017). Lagos University Teaching Hospital(LUTH) is a fee-paying, public-funded tertiary health facility owned by the Federal Government of Nigeria while Massey Street Children’s Hospital is a secondary health facility owned by the Lagos State Government and provides care at no cost to the family. These two health facilities in Lagos receive referrals for young children from all over the country and thus serve as the ideal centers to carry out this study on malnutrition.

The data was collected with the assistance of 2 research assistants who were trained in history taking, physical examination and venipuncture and the data was entered into a standard proforma.

The study participants included children aged 5 months to 5 years with malnutrition according to the WHO criteria for the diagnosis of malnutrition. These children were identified after weight and height were measured. The infants were weighed on a bassinet weighing scale (Docbel Braun Model, Doebil Industries, India) while children above 1 year were weighed on a regularly calibrated scale in light clothing. Weight was measured after checking for zero error with each measurement and the reading was taken to the nearest 0.1 kg. The children were weighed barefooted and in light clothing. All heavy objects and accessories including belt and diapers were removed before weighing. The length of infants was determined with a standard measuring board (Rolla meter mat 100 cm by 1mm) while the height of the older participants was determined to the nearest centimeter with a stadiometer(.(Seca Model, Hardik Medi–Tech Industries, India) Children with weight for height z score of at least <-2 were eligible for participation for the study.

We excluded children with malnutrition and known HIV infection on combination antiretroviral therapy and those whose caregivers did not provide written informed consent.

For each eligible child, we took history of the presenting complaints and conducted physical examination with focus on the presence of fever, diarrhoea, oral thrush, pedal oedema, hepatomegaly, splenomegaly etc.

#### Laboratory tests

In addition to documenting relevant clinical features, we took 5 ml of whole blood from each child for the determination of serum protein, haemoglobin level, white cell count and platelet count and HIV status. In children older than 18 months, HIV test was done using enzyme linked immunosorbent assay (DETERMINE AND UNIGOLD) tests in parallel. When both tests were negative, the result was reported as negative and positive when both tests were positive. When both tests results were discordant, STAT PAK ELISA test was used a tie breaker; if negative the result was reported as negative, and conversely as positive if positive. For children younger than 18 months, HIV status was determined using HIV DNA polymerase chain reaction

The blood samples for full blood count were analyzed using the Mindray five part clinical auto analyzer.

A minimum sample size of 195 children was calculated based on the following assumptions: 12% prevalence of HIV infection in children with malnutrition [12] 95% confidence interval, precision of 0.05 and missing or non-completion rate of the study data entry form of 20%.

Two hundred and thirty five (235) children were initially recruited into the study but 21 excluded due to incomplete data, thus 214 children eventually participated in the study.

### Ethical considerations

We obtained ethical approval from the Health Research Ethics Committee of Lagos University Teaching Hospital before commencement of the study. In addition, all caregivers of study participants provided written informed consent to be eligible to participate in the study. However, verbal consent was obtained from the non- literate caregivers too. We also provided pre and post HIV test counselling to all study participants. The children who tested positive to HIV were referred to the comprehensive HIV clinic in both health facilities for further evaluation and commencement of combination antiretroviral therapy.

#### Definition of terms

Malnutrition was defined using weight for height z score of two or more standard deviation score below the mean or weight for height 70–80% of expected. Using the WHO classification system which recognizes only moderate and severe malnutrition.[13] Severe malnutrition refers to children with weight for height <70%, weight for height three or more standard deviation scores below the mean or any form of malnutrition with peripheral oedema.

The socioeconomic status of each study participant was determined using a commonly employed classification system described by Oyedeji.[14] (Appendix 1) The classification system utilizes the highest educational attainment and occupation of both parents to assign a socioeconomic class. Using this method, class 1 and 2 was designated upper socioeconomic class, 3 middle and 4 and 5 as lower socioeconomic class

1. Moderate malnutrition is weight for height of 70%- 80% of expected or z score of between -2SD to < -3SD.
2. Severe malnutrition refers to any of the following:
  - Weight for height <70% or z score of <-3SD, or
  - Mid upper arm circumference <115mm, or
  - Any form of malnutrition with edema

### Statistical analysis

Data analysis was performed using IBM SPSS Statistics 21.0 (IBM Corporation 2012, USA). Continuous variables were tested for normality of distribution; normally distributed data were summarized using mean and standard deviation while skewed data were summarized as median and interquartile range. Conversely, categorical data were presented as percentages and were compared children with malnutrition with or without HIV infection using student t test or Mann Whitney U test for continuous variables depending on skewness of the distribution, and Chi square test (or Fisher’s exact test as appropriate) for categorical variables. The sociodemographic, clinical and laboratory factors associated with HIV status were determined with the logistic regression analysis.

In all analysis, a p value <0.05 was considered as statistically significant.

## Results

### General characteristics of the study population

A total of 214 malnourished children ≤5 years were recruited into the study, including 160 and 54 children with moderate and severe malnutrition, respectively. Of the 214 study participants 25 (11.7%) tested positive to HIV.

Table 1 shows the general characteristics of the study population in relation to their HIV status. About half (109) of the study participants belonged to the middle socioeconomic class and were Muslims (57.9%). Children with malnutrition and HIV infection were similar in age and weight for height z score, gender distribution with children with malnutrition without HIV infection. Also, both groups of children had similar weight for height z score.

**Table 1:** General characteristics of the study participants.

Parameter	Children with HIV, n = 25	Children without HIV, n = 189	Odds ratio(95% CI)	p value
Age, median (IQR), months	12(9–36)	12 (5–60)	-	0.78
Age category (months)				
<12	5(8.2)	56(91.8)	0.68(0.27–1.80)	0.49
12–24	15(12.1)	109(87.9)	1.10(0.47–2.57)	0.82
25–36	3(25)	9(75)	2.72(0.68–10.83)	0.15
>36	2(11.8)	15(88.2)	1.01(0.21–4.69)	1.00
**Gender, n (%)**				
Male	11 (44.0)	83 (43.9)	1.00 (0.43–2.32)	0.99
**Female**	14(56.0)	106(56.1)	1.00(0.43–2.32)	0.99
**Weight,** median (IQR) Kg	7.7 (6.5–8.5)	7.7 (6.00–8.10)	-	0.78
**Length/Height,** median (IQR), cm	69.7 (64.4–75.0)	70.2 (65.0–76.0)	**-**	0.36
Weight for height z score	-2.7 (-4.0to-2.0)	-2.5 (-3.7 to -2.0)	**-**	0.22
Length or Height for age z score	-2.8 (-3.4to -0.95)	-2.9 (-3.63 to -0.46)		0.87
**Maternal age** median (IQR), year	29 (26–34)	30 (25–33)	**-**	0.92
**Socioeconomic status N (%)**				
High	1(2.7)	36(97.3)	0.17(0.02–1.35)	0.08
Middle	14(12.8)	95(87.2)	1.25(0.54–2.91)	0.59
Low	10(14.7)	58(85.3)	1.50(0.63–3.55)	0.34
**Religion N (%)**				
Christianity	12(16.4)	61(83.6)	1.94(0.85–3.71)	0.17
Islam	12(9.7)	112(90.3)	0.63(0.27–1.46)	0.28
Others	1(6.3)	16(93.7)	0.45(0.05–3.55)	0.69
**Malnutrition Classification**				
**-2SD to -3SD**	19(11.9)	141 (88.1)	1.07 (0.41–2.86)	0.89
**>-3SD**	6(11.1)	**48(88.9)**		

N%- number(percentage), CI-Confidence Interval, IQR- Interquartile range

#### HIV seropositivity

Twenty five of the malnourished children were positive for HIV, thus the prevalence of HIV in the subjects with was 11.7%. The lowest seropositivity was seen in the infants (8.2%) as shown in Table 1. Although, fewer children (2.7%) with malnutrition belonging to families of high socioeconomic class had HIV infection, the difference was not statistically significant when compared with those from middle (12.8%) and low (14.7%) classes. Similarly, although children less than 12 months old with malnutrition had the lowest prevalence (8.2%) of HIV infection the difference was not significant when compared with other ages.

Children with moderate and severe malnutrition had similar HIV prevalence of 11.9% and 11.1% (P = 0.89), respectively. (-2.51 vs -2.50)

### Clinical features

Fever, cough and diarrhea were the most common symptoms seen amongst the study participants while oral thrush, dermatoses and splenomegaly were the most common signs. (see Table 2). A higher proportion of the HIV positive subjects compared to the negative subjects had oral thrush, dermatoses and edema but this was not statistically significant. Hepatomegaly and splenomegaly were was the least presenting signs in both groups.

**Table 2:** Clinical characteristics of the malnourished children: HIV positive and HIV negative children.

Clinical features	HIV Positive(N = 25)	HIV Negative(N = 189)	P value
Fever	16(64)	119(62.9)	0.92
Cough	13(52)	92(48.7)	0.75
Oral Thrush	3(12)	8(4.2)	0.09
Diarrhea	5((20)	56(29.6)	0.32
Jaundice	0	2(1.1)	0.61
Edema	2 (8)	6 (3.2)	0.23
Dermatoses	3(12)	17(8.9)	0.63
Hepatomegaly	2(8)	12(6.3)	0.75
Splenomegaly	2(8)	0(0)	0.00

HIV-Human immunodeficiency virus

None of the subjects with HIV had otitis media or Jaundice. Splenomegaly was found in 2 (8.0%) children with malnutrition and HIV compared with 0 (0%) in the group with malnutrition without HIV, Splenomegaly was the only significantly different sign among the 2 groups of malnourished children (p = 0.00).

Table 3 shows the laboratory investigations in the study participants. The Packed cell volume (PCV) ranged between 14.1% - 52.5% with a median (IQR) of 35.2% (28.7–38.1) in the study participants.

**Table 3:** The laboratory investigations in the study participants.

Parameter	Reference value	HIV positive Median(IQR)	HIV negative Median(IQR)	P value
PCV(%)	34.0–40.0	34.7 (28.77–38.10)	35.4 (32.0–33.0)	0.39
Total WBC(x10^9^/L)	4.0–11.0	11.1 (9.47–15.95)	11.80 (9.02–16.17)	0.60
Neutrophils (%)	25.0–57.0	47.4 (28.30–62.00)	47.8 (34.80–59.90)	0.75
Lymphocytes (%)	25.0–54.0	41.40 (27.60–57.30)	41.65 (29.72–54.77)	0.85
Platelets(x10^9^/L)	150,000–450,000	431,000 (294,000–494,000)	445,000 (333,000–586,000)	0.15
Total protein(g/dl)	57.0–80.0	66.4 (63.1–73.6)	63.5 (58.5–68.3)	0.15
Serum Albumin(g/dl)	35.0–55.0	36.2 (28.6–40.8)	37.2 (31.4–41.0)	0.66
Serum Globulin(g/dl)	25.0–45.0	30.53(24.61–39.73)	27.5 (23.3–31.2)	0.11

HIV-Human immunodeficiency virus, PCV-Packed cell volume, WBC- White blood cell count. IQR- Interquartile range.

The median PCV was comparable in both groups. 30(14%) of the study participants had anaemia of which only 2 (0.9%) of the subjects had severe anaemia. PCV less than 20% was regarded as severe anaemia.

Total white blood count (WBC) ranged from 1,630–56,600cells/mm3 with a median of 11,600/mm3. The WBC and platelets were lower in the HIV positive subjects than the HIV negative subjects but this did not reach statistically significant levels. (p = 0.15) The proportion of the neutrophil and lymphocytes were also comparable in the two groups.

The serum globulin levels were higher in the HIV positive group but this did not reach statistically significant levels too (p = 0.11). Similarly, the serum albumin though lower in the HIV positive group did not reach statistically significant levels. (p = 0.66) CD4 count and CD4% in the HIV group were 645.00 (293.00–1,060) and 12.25% (10.00–14.25) respectively.

Multivariate analysis was used to evaluate the risk factors for HIV in the study participants and this is shown in Table 4. Multivariate analysis showed that none of the factors could significantly predict the occurrence of HIV in the study population.

**Table 4:** Predictors of the risk of occurrence of HIV in the malnourished children: Logistic regression.

Parameter	Odd’s ratio (95% CI)	P value
Age(months)		
<12 versus > 36	2.60×108(0.00)	1.00
12–36 versus >36	1.53×108 (0.00)	1.00
Gender	1.13 (0.29–4.4)	0.86
Socioeconomic status		
Low versus High	5.62(0.63–49.7)	0.27
Middle versus High	4.49(0.52–38.56)	0.61
Nutritional status		
Severe versus Moderate	1.99(0.56–6.99)	0.99
Oral thrush	2.69(0.56–12.89)	0.21
Globulin	1.01(0.99–1.04)	0.14
PCV	1.08(0.97–1.22)	0.15
Platelet count	0.99(0.99–1.00)	0.98

P< 0.05 is considered significant, PCV- Packed cell volume, CI-Confidence Interval

## Discussion

In this study, the prevalence of HIV infection amongst malnourished children aged 5 years and below attending two health facilities in Lagos was 11.7%. This prevalence is much higher than the national prevalence for HIV in Nigeria (4.4%) and from other reports in malnourished children in the country.[14–16] The possible reasons for the high prevalence observed in this present study may be due to the fact that this was a hospital based study and a smaller cohort of children presenting to the health facilities were studied compared to the some of the previous studies which were community based. In addition, some of the previous studies only evaluated children with severe malnutrition only. Ignorance, weak PMTCT services and failure of early infant diagnosis in the country may contribute to the findings in this present study but these latter factors were not explored in the present study.

The highest rate of seropositivity (25%) was seen in the 25–36 months age group. This age bracket was the peak age of presentation of the infection and probably represented the second phase in HIV infection. It has also been observed that the children who did not receive treatment at this time are likely to die from the illness.[17] Currently, most children who receive treatment and are compliant now survive into adolescent period and beyond.[15]

The use of the WHO classification for malnutrition actually reflects the severity of the malnourished state. According to this classification majority of the subjects in this present study had moderate malnutrition and the severe forms was seen in a quarter of the subjects. Edematous malnutrition was present in minority (3.7%) of the subjects. Other reports from south western Nigeria[18,19] have also observed that the less severe forms of malnutrition are less prevalent in south western Nigeria but still remains a problem in northern Nigeria.[18,19] Recent reports as documented in the global nutrition impact[20] reflect that increasing global efforts has led to effective reduction in severe forms of malnutrition in some countries including the sub-Saharan African countries, National efforts, such as advocacy for exclusive breastfeeding, appropriate complementary feeding etc in Nigeria may also possibly explain the observation of the reduced prevalence in the less severe forms of malnutrition observed in the south western Nigeria too.[20, 21]

Majority of the HIV positive subjects in this present study also had moderate malnutrition since this was commonest form of malnutrition seen in the subjects. In contrast, Sudawa et al[17] in 2013 in Kano state observed that marasmus (48.3%) was the commonest type of malnutrition seen in underfives with HIV infection but as observed in our study only minority (6.5%) had edematous malnutrition. Severe wasting without edema is a common finding in severe malnutrition with concomitant HIV infection.[22–25] Presently, WHO has corroborated this fact by classifying wasting as seen in Marasmus as a stage 3 AIDS defining illness. Thus, the need arises to always screen children with severe wasting for retroviral disease.

Many studies on malnourished children and HIV status have focused on severely malnourished children as the target group for HIV and the possible reason may be related to the difficulty in differentiating the two conditions. This present study is one of the few reports [14–15,17] which documents the retroviral status in both moderately and severely malnourished children in Nigeria, however being a hospital based study the results cannot be readily extrapolated to the general population.

In terms of clinical findings, fever, cough and diarrhea were the commonest symptoms seen in the subjects in this present study. These findings are similar to that of previous workers.[15,17,19]Splenomegaly was seen more in the HIV subjects and appeared to be the only discriminatory clinical feature amongst the HIV positive and negative malnourished children. Severe malnutrition and HIV may be difficult to differentiate clinically especially in the resource limited countries however some authors have noted that the severely malnourished HIV infected children were more likely to have oral thrush and persistent diarrhea and these may serve as possible discriminating features.[3,20–21,23–26]

The haematologic indices evaluated in the malnourished children were observed to be lower in the HIV positive group compared with the HIV negative group but this was not statistically significant. This is consistent with the findings of Ezeonwu et al [27] in Enugu and Adetifa et al[28] in Lagos. Malnourished children are predisposed to anaemia for several reasons namely inadequate intake, micronutrient deficiency (iron and folate deficiencies), increased nutrient loss from recurrent diarrhea. These conditions are prevalent in the HIV infected children and these factors may influence the prevalence of anemia in this group of children. [28]

The total protein in the HIV group in this present study was higher than the HIV negative group but the difference was not statistically significant. A higher level of serum proteins in HIV infection has been reported by other workers[27–29] and this finding has been attributed to hyperglobulinaemia. In our study the globulin level was also found to be higher in the HIV positive subjects compared to the HIV negative children. Theories postulated for the hyperglobulinaemia is the polyclonal B cell activation.[27–29] The viral proteins (glycoprotein 41) is said to be responsible for the activation of the polyclonal B cell which occurs as the infection progresses.[27–29]However, the serum albumin was lower in the HIV group compared to the non HIV group and this may still be a reflection of the malnourished state rather than the HIV infection.

The predictors of the occurrence of HIV in the malnourished subjects were determined with the use of the multivariate analysis but none of the factors examined could significantly predict the risk of occurrence of HIV infection in this cohort of malnourished children. Reported predictors/risk factors in malnourished children by Madec et al[10] however includes gender and previous hospitalizations.

### Limitations

This was a hospital based study with a relatively small sample size and thus it may be difficult to generalize the results of the findings of the study. It would have been desirable to follow up the subjects and monitor any changes in the haematological and biochemical profile but this was difficult due to logistic reasons.

## Conclusions

Malnourished children remain a high risk group for HIV infection and the prevalence of the infection obtained in this group of children is still unacceptably high. Discriminatory features between malnutrition and HIV remains difficult. The presence of hyperglobulinaemia on laboratory analysis in a malnourished child may heighten the suspicion of possible underlying associated HIV infection. Screening of malnourished children for HIV infection and further longitudinal studies on malnourished children with HIV is advocated.

## APPENDIX I

### OYEDEJI’S CLASSIFICATION

Education

1. University graduate/equivalent
2. School Certificate/ Teaching or other professional training
3. School Certificate or Grade II Teacher’ Certificate or
4. Modern 3 or Primary 6
5. Barely literate or illiterate

Occupation

1. Senior public servant, professional, manager large scale trader, business entrepreneur and contractors
2. Intermediate grade public servant and senior secondary school teachers
3. Junior secondary school teachers, drivers and artisans
4. Petty traders/ labourers, messengers and other similar grades
5. Unemployed, full time homemaker, student and subsistence farmers

## Supporting information

S1 Database Database for the study. (SAV)
